# Lamellar Keratoplasty Using Acellular Bioengineering Cornea (BioCorneaVet^TM^) for the Treatment of Feline Corneal Sequestrum: A Retrospective Study of 62 Eyes (2018–2021)

**DOI:** 10.3390/ani12081016

**Published:** 2022-04-13

**Authors:** Huihao Xu, John S. Sapienza, Yipeng Jin, Jiahao Lin, Xiaobo Zheng, Haodi Dong, Hongxiu Diao, Ying Zhao, Jiafeng Gao, Jing Tang, Xueqian Feng, Danielle Micceri, Haoran Zeng, Degui Lin

**Affiliations:** 1 College of Veterinary Medicine, China Agricultural University, No. 2, Yuanmingyuan West Road, Haidian District, Beijing 100193, China; xuhuihao2dai@163.com (H.X.); cauvet1@cau.edu.cn (Y.J.); jiahao_lin@cau.edu.cn (J.L.); dhd0905@cau.edu.cn (H.D.); diaohongxiu@yeah.net (H.D.); zying4405@163.com (Y.Z.); caugaojiafeng@163.com (J.G.); 2 College of Veterinary Medicine, Southwest University, No. 160, Xueyuan Road, Rongchang District, Chongqing 402460, China; oppyp@163.com (X.Z.); tangjing147@email.swu.edu.cn (J.T.); fxq195357@email.swu.edu.cn (X.F.); haoranzeng@163.com (H.Z.); 3 Long Island Veterinary Specialists, Plainview, NY 11803, USA; jsapeye@gmail.com (J.S.S.); danielle.micceri@medvet.com (D.M.)

**Keywords:** acellular bioengineering cornea, corneal sequestrum, keratectomy, feline, xenograft, optical coherence tomography

## Abstract

**Simple Summary:**

Corneal sequestrum is a specific and common corneal disease in cats. Surgery treatment is the recommended option. Acellular bioengineering cornea (ABC) is a popular and effective corneal transplantation material. However, no study has been published to evaluate the effectiveness and outcome of ABC lamellar transplantation for the treatment of feline corneal sequestrum (FCS). The purpose of this retrospective study was to evaluate the surgical effect of ABC lamellar transplantation in the treatment of FCS. All cats were diagnosed with FCS. All eyes received ABC lamellar transplantation for the first time, including 61 cats (62 eyes), aged 6–120 months. The average sequestrum size was 7.98 mm, with a medium of 7.75 mm (range, 4.75–11.75 mm), and the sequestrum thickness included 200 microns for 1 eye (1.61%), 300 microns for 28 eyes (45.16%), 400 microns for 29 eyes (46.77%), and 450 microns for 4 eyes (6.45%). All eyes retained vision after surgical treatment, and there was no recurrence during the follow-up period. This study has several limitations, including incomplete unification and standardization of data collection, some vacancies of follow-up time, inconsistency between then optical coherence tomography(OCT) examination and postoperative photo collection. Despite several limitations, our results show that ABC is easy to obtain and store, and has the choice of different sizes and thicknesses to achieve rapid corneal healing, and satisfactory visual and cosmetic effects in FCS treatment. Acellular bioengineering cornea can be a good alternative for the treatment of FCS.

**Abstract:**

To retrospectively evaluate the effectiveness and outcome of lamellar keratoplasty using acellular bioengineering cornea (BioCorneaVet^TM^) for the treatment of feline corneal sequestrum (FCS). The medical records of cats diagnosed with FCS that underwent lamellar keratoplasty with BioCorneaVet^TM^ between 2018 and 2021 with a minimum of 3 months of follow-up were reviewed. Follow-up examinations were performed weekly for 3 months, and then optical coherence tomography (OCT) examination was performed on select patients at 0, 3, 6, and 12 months post-operatively. A total of 61 cats (30 left eyes and 32 right eyes) were included. The Persian breed was overrepresented, 48/61 (78.69%). Four different thicknesses of acellular bioengineering cornea were used (200, 300, 400, or 450 microns), and the mean graft size was 8.23 mm (range, 5.00–12.00 mm). Minor complications were composed of partial dehiscence, and protrusion of the graft occurred in 7/62 eyes (11.29%). The median postoperative follow-up was 12.00 months (range, 3–41 months). A good visual outcome was achieved in 60/62 eyes (96.77%), and a mild to moderate corneal opacification occurred in 2/62 (3.23%). No recurrence of corneal sequestrum was observed. From the results, lamellar keratoplasty using acellular bioengineering cornea (BioCorneaVet^TM^) is an effective treatment for FCS, providing a good tectonic support and natural collagen framework, and resulting in satisfactory visual and cosmetic effects.

## 1. Introduction

Corneal sequestrum is a specific and common corneal disease in cats, particularly in the Persian and Himalayan breeds [1,2]. The condition is thought to be associated mainly with chronic corneal irritation or ulcer, iatrogenic trauma, and feline herpesvirus-1 (FHV-1) [1,3,4,5]. Feline corneal sequestrum (FCS) is characterized by brown-to-black plaques, which can present stromal depths ranging from superficial to a very deep corneal lesion that may encompass all layers of the corneal stroma rostral to Descemet’s membrane. The lesion may protrude with vascular invasion, corneal edema and inflammation, and in the most serious cases, leading to deep corneal ulcer or even perforation. The affected eye may suffer from reduced vision, blindness, or even loss of the globe.

For the treatment of corneal diseases in both humans and animals, the primary aim is to sustain corneal integrity and restore transparency to the greatest possible extent. For many cases of FCS, surgery is an important treatment option. A number of surgical methods and materials are currently available for the treatment of such a disorder, including lamellar keratectomy combined with conjunctival grafts [1], porcine small intestinal submucosa (BIOSIS) transplantation [6,7], porcine bladder ACell^®^ tissue graft transplantation [8], corneo-conjunctival transposition (CCT) [2,9], bovine pericardium graft [10], cyanoacrylate adhesive [11], amniotic membrane transplantation [12], and homograft or xenograft corneal transplantation [13,14,15].

Most of these surgical methods or materials do not provide the corneal clarity compared with a freshly harvested corneal allograft. The latter, however, is difficult to apply extensively in veterinary medicine due to the scarcity of donor sources and preservation technologies for the feline patient. Acellular biomaterials are characterized by retained natural extracellular matrix, inactivated antigens and pathogens, and their structures are more conducive to tissue remodeling. Therefore, acellular biomaterials to repair corneal tissue defects have been extensively studied in recent years [16,17].

The acellular bioengineering cornea (ABC) material considered in this study, BioCorneaVet™ (Sanhe PetsEyes Pets Foods & Products Co., Ltd., Langfang, China), consists of an extracellular collagen matrix (ECM) derived from porcine cornea. The tissue, procured through a method of viral inactivation, decellularization, and sterilization (gamma radiation), can be stored for up to 18 months at a temperature between 2–25 °C. The acellular porcine corneal stroma is prepared by a series of controlled procedures, including the removal of antigens such as heterogeneous cells, miscellaneous proteins, and polysaccharides from the cornea whilst retaining the natural corneal matrix collagen scaffold [18].

To the best of the author’s knowledge, there are no publications describing the use of ABC combined with lamellar keratoplasty for treatment of FCS in a significant number of cases. The aim of this retrospective study was to describe the use of this ABC combined with lamellar keratoplasty for the surgical treatment of FCS, and to evaluate postoperative tectonic and visual effects.

## 2. Materials and Methods

### 2.1. Animals, Data Collection, and Preoperative Treatment

Animals were admitted to the Teaching Animal Hospital of China Agricultural University and the Teaching Animal Hospital of Southwest University between February 2018 and October 2021. Data were collected and reviewed from case records. All animals underwent complete ophthalmic examinations including evaluation of menace response, dazzle reflex, pupillary light reflex, palpebral reflex, Schirmer tear test (STT I) (Eickemeyer, Tuttlingen, Germany), fluorescein staining (Entod Research Cell UK Ltd., London, UK), slit-lamp biomicroscopy (KOWA SL-17, KOWA Co., Ltd., Tokyo, Japan), indirect ophthalmoscopy examination (Keeler Advantage Plus, Malvern, PA, USA), and intraocular pressure measurement (Tonovet Plus, Icare Finland Oy, Vantaa, Finland). The performance of polymerase chain reaction (PCR) testing for feline herpesvirus-1 was completed in 21 cases. A thorough physical examination, complete blood cell count, and serum biochemistry were performed on all animals. The relevant data from the medical records were collected: breed, gender, age, affected eye, ocular diseases, treatment history, location and degree of sequestrum, size and depth of graft, outcome, and consent form from the owner, and Ethics Committee approval. Only patients with a minimum follow-up of three months were included in the present study.

Preoperative administration included topical 0.5% levofloxacin eye drops (Nitto Medic Co., Ltd., Toyama, Japan) four times daily, 1% atropine gel (SINQI^®^, Sinqi Pharmaceuticals, Shenyang, China) once daily and 0.3% sodium hyaluronate drops (I-DROP^®^ VET GEL, I-MED ANIMAL HEALTH SANTÉ ANIMALE, Saint-Laurent, QC, Canada) six times daily for cats, additionally 0.1% idoxuridine eye drops (Meds for Vets, LLC, Sandy, UT, USA) six times daily and famciclovir (FAMVIR^®^, Novartis Farmaceutica S.A., Barcelona, Spain) 90 mg/kg BID were prescribed for oral use in those cats with herpes virus-1 infection.

### 2.2. Surgical Procedure

Before surgery, information about the lesions was obtained by optical coherence tomography (OCT) (OSE-2000AS, Hilton Technology Co., Ltd., Shenzhen, China) to help formulate the operation plan (mainly the specific parameters, including the fixation mode of internal and external fixation, 840-nm super luminescent diode and corneal L-Scan pattern). General anesthesia was induced with dexmedetomidine (IM) (DEXDOMITOR^®^, Zoetis, Parsippany, NJ, USA) 5 μg/kg, and propofol (IV) (Guangdong Jiabo Pharmaceutical Co., Ltd., Qingyuan, China) 2 mg/kg, and maintained with isoflurane anesthesia (JIANGSU ZHONGMU BEIKANG PHARMACEUTICAL Co. Ltd., Taizhou, China). All cats were positioned in dorsal recumbency with vacuum headrest. The affected eyes were prepared with 1% Povidone-iodine solution for antisepsis.

**Figure 1:**
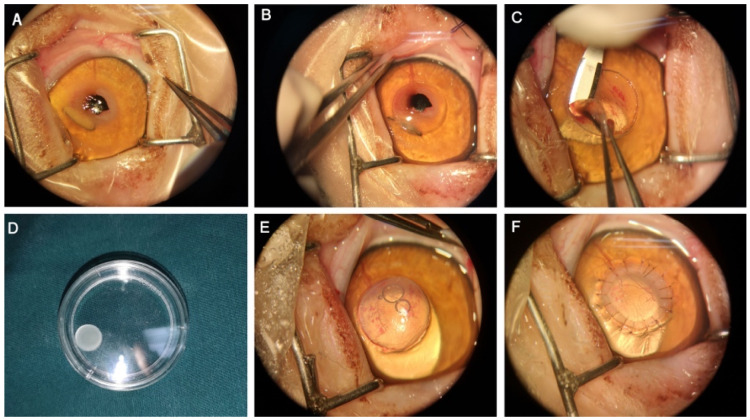
Surgical procedures of lamellar superficial keratectomy with BioCorneaVet^TM^ (Case 1). (**A**) The affected eye was prepared for surgery and the sequestrum was measured; (**B**) A keratectomy was performed to the corneal sequestrum with a corneal trephine; (**C**) A crescent blade was used to completely remove the sequestrum; (**D**) An appropriate graft (400 microns thickness) was selected; (**E**) The graft was trephined to the appropriate diameter (7.50 mm); (**F**) The graft was positioned by simple interrupted absorbable sutures.

All operations were performed with the aid of an operating microscope (CORELLA XY/FS1-12, Moeller, Wedel, Germany). Briefly, the size of the lesion was measured with calipers under the operating microscope, and then a trephine 0.25 mm larger than the diameter of the lesion was selected to perform lamellar keratotomy (Figure 1A,B). A crescent blade (Mani Inc., TOCHIGI, Japan) was used to perform lamellar dissection until the lesion was completely removed (Figure 1C). Depending on the thickness of the corneal excised stroma, an applicable size of sterile BioCorneaVet^TM^ (Sanhe PetsEyes Pets Foods & Products Co., Ltd., Langfang, China) was applied (200, 300, 400, or 450 microns were used in this study) (Figure 1D). Following the manufacturer’s instructions, the BioCorneaVet^TM^ was rehydrated with normal saline for 10 s, no longer than 30 s, and then a matching graft was obtained by cutting with a trephine 0.25 mm larger than the corneal defect (Figure 1E). The graft was sutured into the implant bed using an interrupted suture with 9/0 absorbable polyglycolic (USIOL Inc., Lexington, KY, USA), or a 10/0 non-absorbable nylon (Mani Inc., TOCHIGI, Japan) suture in some cases in order to strengthen the fixation (Figure 1F). During the operation, irrigation of the ocular surface with balanced salt solution was performed to prevent excessive desiccation of the cornea, but less frequently than usual, so as not to accelerate the edema of the graft. For the cases with simultaneous entropion, the correction of entropion was performed with modified Hotz–Celsus procedure first. The corneal erosion was cleaned and debrided with a sterile cotton swab, and the bullous lesions were treated with thermoplasty. OCT examination was performed again immediately after the operation to evaluate the implant status. Subjects were advised to wear an Elizabethan collar for a minimum of 2 weeks after surgery.

Postoperative treatment was initiated immediately after the operation and consisted of topical administration of 0.5% levofloxacin eye drops 6 times daily for 1–3 weeks, 0.3% sodium hyaluronate eye drops 6 times daily for 8 weeks, and 1% atropine gel once daily for 1 week. Meloxicam (Shanghai Hanwei Biomedical Technology Co., Ltd., Shanghai, China) 0.1 mg/kg/day was prescribed orally for 5 days. Topical antibiotics were not discontinued until the corneal re-epithelization was achieved, through the assessment of a negative fluorescein test. Once corneal epithelialization was completed, 1% cyclosporine (North China Pharmaceutical Co., Ltd., Shijiazhuang, China) twice daily were used to help the reduction of corneal vascularization and scarring/fibrosis.

### 2.3. Follow-Up

Post-operative examinations of all cats were performed at 1, 2, 3, 4, 5, 6, 7, and 8 weeks, and then at 3-, 6-, and 12-months after surgery. In some cases, the frequency of post-op examination was much higher because of requests from the owners. A complete ophthalmic examination was performed including dazzle reflex, menace response, STT I, intraocular pressure measurement, fluorescein staining, slit-lamp biomicroscopy, and indirect ophthalmoscopy. In addition, OCT was performed at 3-, 6-, and 12-months post-surgery to evaluate the structure and morphology of the grafts and corneas. A postoperative vision evaluation system by Chow et al., 2016 [8], was used in this study, illustrating that cases were divided into five categories, based on the presence of corneal blood vessels, the opacity of the corneal scar, the positive menace response, the ability to visualize the posterior and/or the anterior segment through the grafted area at their last examination, and the time period from the surgery to the last examination was at least 3 months. Questionnaires were completed with 60 owners to evaluate satisfaction with the outcome of the surgery.

### 2.4. Statistical Analysis

The categorical variables (breed, gender, affected eye, ocular abnormalities, location of sequestrum, graft thickness, corneal opacification, and complications) are presented as percentages, while the continuous variables (age, graft size, and re-epithelization time) are presented as means or medians and the range.

## 3. Results

### 3.1. Animals

Sixty-two eyes in sixy-one cats with FCS were included in this study (Table S1). The Persian breed was overrepresented (*n* = 48, 78.69%), followed by British Shorthair (*n* = 6, 9.84%), American Shorthair (*n* = 4, 6.56%), domestic shorthair (*n* = 1, 1.64%), Ragdoll (*n* = 1, 1.64%), and Munchkin (*n* = 1, 1.64%). There were forty-three males (70.49%) and eighteen females (29.51%) with the ages ranging from 6 months to 120 months (median age 29.50 months), including twenty-four (39.34%) castrated males and eleven (18.03%) spayed females. Left eyes (*n* = 30, 48.39%) and right eyes (*n* = 32, 51.61%) were affected. In all eyes, sequestrum was mainly located at the axial cornea (*n* = 47, 75.81%) or paracentral (*n* = 15, 24.19%). Almost all eyes also had corneal neovascularization and varied degrees of inflammation, although the size and depth of sequestrum varied. Twenty-one of the cats (34.43%) were tested for FHV-1 by PCR during the initial examination, and eleven of them were positive (52.38%). Nineteen eyes had concomitant eyelid and/or corneal abnormalities (30.65%), included eyelid entropion (*n* = 4, 6.45%), bullous keratopathy (*n* = 9, 14.52%), corneal lipid deposition (*n* = 1, 1.61%), corneal melting (*n* = 1, 1.61%), corneal epithelial erosion (*n* = 5, 8.06%), and scar after conjunctival flap (*n* = 1, 1.61%).

### 3.2. Clinical Results and Complications

**Figure 2:**
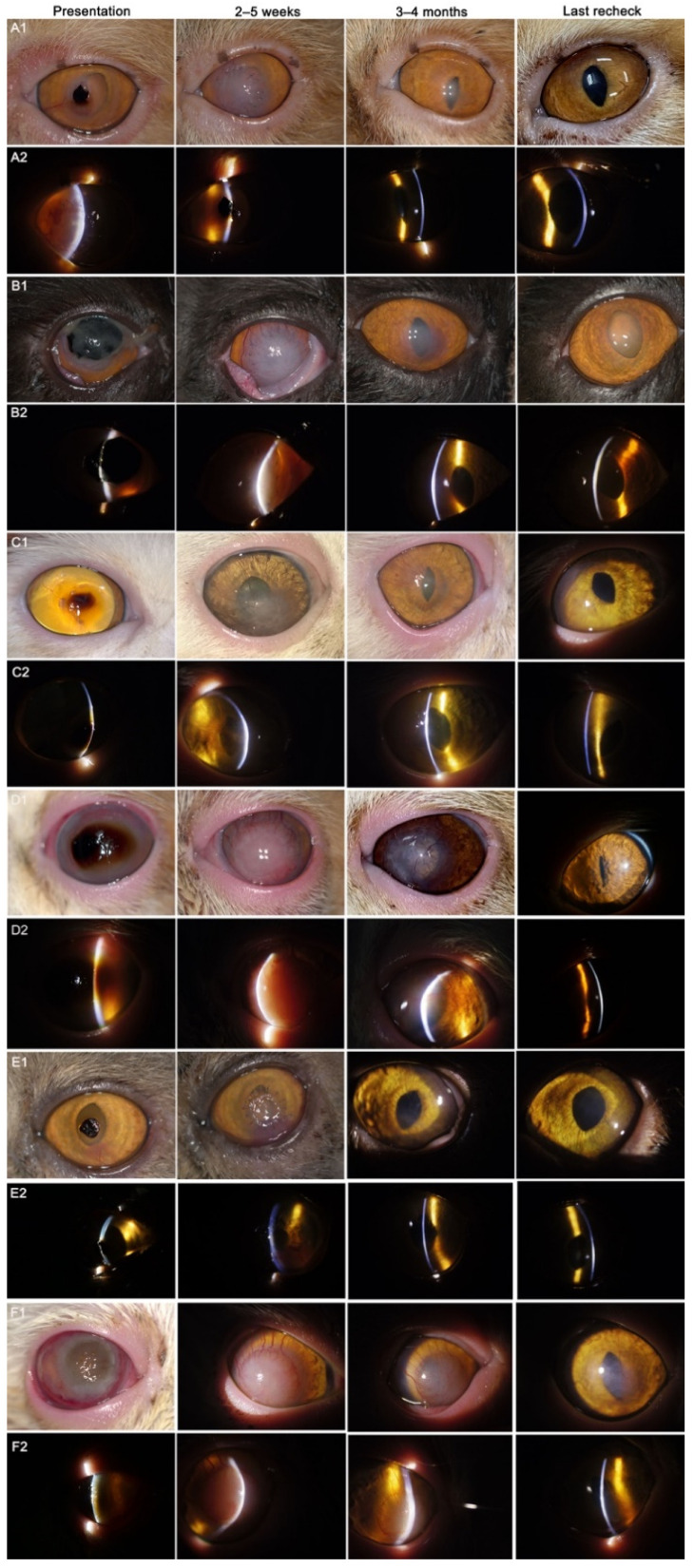
Photographic records of some cases were included in the present study. The first column is at presentation; the second column is 2–5 weeks after keratoplasty; the third column is 3–4 months after keratoplasty; and the fourth column is at the last recheck. (**A1**, **B1**, **C1**, **D1**, **E1** and **F1**) By camera; (**A2**, **B2**, **C2**, **D2**, **E2** and **F2**) By slit-lamp biomicroscopy; (**A1**–**F2**) Case number: 1, 2, 6, 18, 24 and 53.

**Figure 3:**
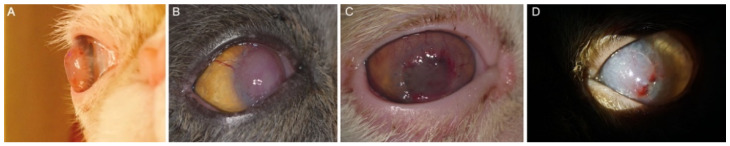
Potential complications after acellular bioengineering cornea lamellar keratoplasty. (**A**,**B**) Protrusion of the graft (3–4 weeks post-op); (**C**) Obvious vascularization of the graft (2–3 weeks post-op); (**D**) Partial dehiscence of the graft (2 weeks post-op); (**A**–**D**) Case number: 6, 12, 53 and 29.

**Figure 4:**
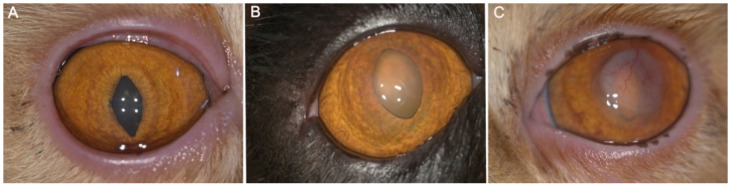
Grading of corneal healing after BioCorneaVet^TM^ lamellar keratoplasty. (**A**) Grade 0, almost invisibility of corneal scarring and vessels; (**B**) Grade 1, minimal corneal stroma opacity and vascularization; (**C**) Grade 2, mild to moderate corneal stroma opacity and vascularization; (**A**–**C**) Case number: 3, 2 and 8.

**Figure 5:**
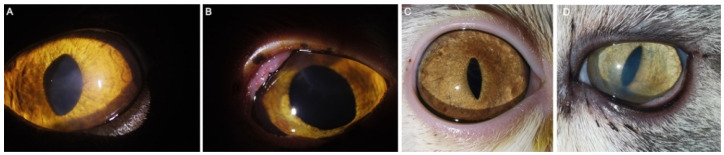
Good and satisfactory examples of functional and cosmetic outcomes. (**A**–**D**) Case number: 11 (24 months post-op), 26 (14 months post-op), 32 (6 months post-op) and 33 (11 months post-op).

In the present study, all cats received lamellar keratoplasty for the first time. The selection of graft thickness and diameter depends on the sequestrum size and depth. The average graft size was 8.23 mm, with a medium of 8.00 mm (range, 5.00–12.00 mm), and the graft thickness included 200 microns for 1 eye (1.61%), 300 microns for 28 eyes (45.16%), 400 microns for 29 eyes (46.77%), and 450 microns for 4 eyes (6.45%). All eyes were sutured with 9/0 absorbable suture, some extra sutures of 10/0 nylon were added for two eyes only to strengthen the fixation. Complete re-epithelization of the grafts was obtained after surgery by 5–15 days in all eyes, with a mean of 7.39 days and a medium of 7.00 days. Sixty-two grafts were eventually incorporated into corneas (Figure 2). The grafts were slight edematous in five eyes (8.06%) 1-week postoperatively, gradually thickened and protruded for 3–4 weeks thereafter (Figure 3A,B). The protrusion of the graft in most cases was unable to be seen between the second and third month postoperatively, according to the variety of the graft and postoperative cell infiltration, depending on the type of graft and postoperative cell infiltration. Corneal vascularization started to appear from the second or third week after surgery and gradually migrated towards the graft (Figure 3C). Vascularization gradually improved with topical immunosuppressant agents in 2–3 months. The grafts showed various degrees of vascularization with a large number of cell infiltrations according to individual variation during the 4–16 weeks post-surgery. Nylon sutures, if placed, were removed at this time. Partial dehiscence of the sutures occurred in two eyes (3.23%) (Figure 3D). Surgical closure of the dehisced area was performed in these two eyes. The transparency of the cornea was typically restored by the third or fourth month after surgery, and grafts were almost transparent in the sixth month, with mostly ghost vessels visible with slit-lamp biomicroscopy. Although minor complications, including partial dehiscence and protrusion of the graft, occurred in seven eyes (11.29%) post-operatively, it was shown that on the last recheck, stable corneal integrity with the retained vision was obtained with the outcome of the surgery (corneal appearance and visual function) in all 62 eyes, consisted of grade 0 in 50 eyes (80.65%), grade 1 in 10 eyes (16.13%) and grade 2 in 2 eyes (3.23%) (Table 1, Figure 4). No recurrence of corneal sequestrum was observed in any operated cats. The mean postoperative follow-up was 14.65 months, with a median of 12.00 months (range, 3–41 months). A total of 56 eyes (90.32%) had a follow-up time of 6 months at least, in which 22 eyes (35.48%) between 6 and 12 months, and 34 eyes (54.84%) were more than 12 months (including 12 months) follow-up time, respectively. All owners involved in the questionnaire (60/62, 96.77% owners were subject to the questionnaire) were satisfied with the outcome (corneal appearance and vision) of the surgery (Figure 5).

### 3.3. Optical Coherence Tomography Results

**Figure 6:**
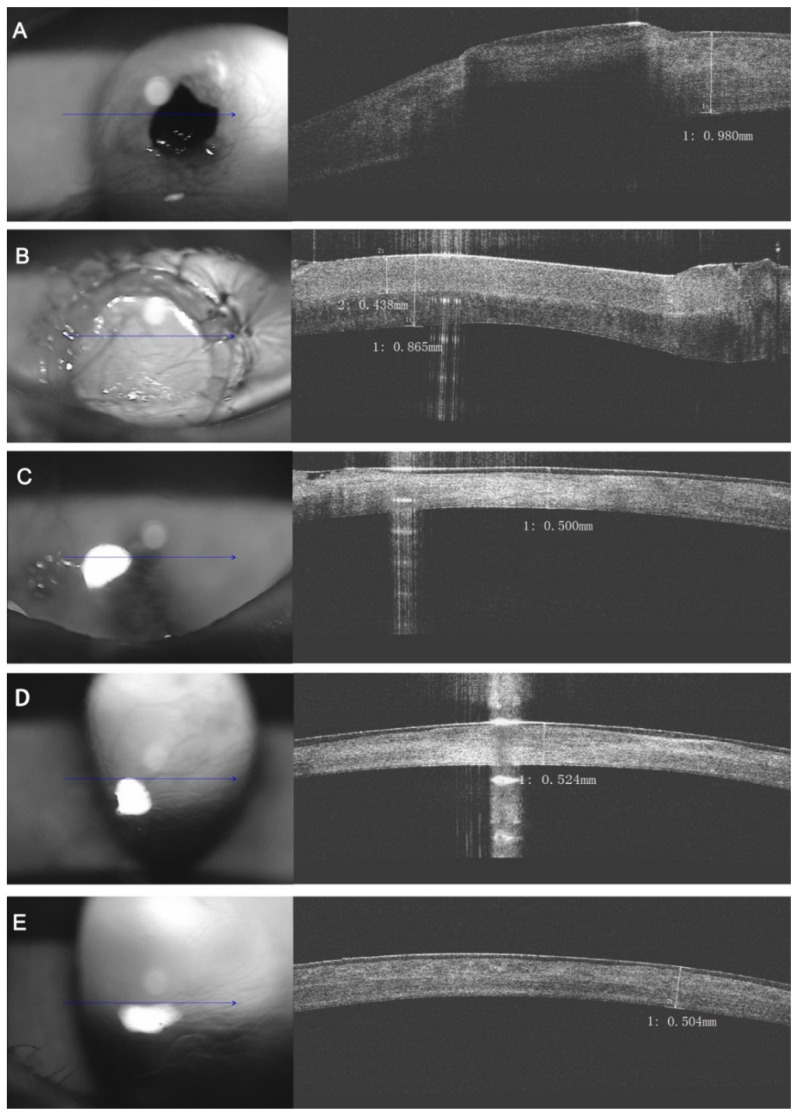
The same patient as Figure 1. Optical coherence tomography (OCT) upon the eye. (**A**) Preoperative image; (**B**) Immediate examination after acellular bioengineering cornea lamellar keratoplasty: The fine interface between the graft and the recipient side; (**C**) Three months post-operatively: No obvious transition between the graft and the recipient side, both of which were stable; (**D**) Six months post-operatively: The whole cornea was showed highly integral and uniform; (**E**) Twelve months post-operatively: The alignment of the collagen fibers in the whole cornea was integrated and restored. (Blue arrows: corneal L-Scan pattern).

A total of 15 eyes underwent OCT examination; other eyes were not examined due to the cost of the procedure, which the owners refused to perform. The depth of sequestrum in all 15 eyes reached at least half of the corneal thickness (Figure 6A). Immediately after the surgery, the OCT image showed the graft had a good attachment with the implant bed. The graft surface was smooth and the places of suture knots on the graft were intact and integrated. The density of the graft was observed as obviously thicker than the recipient’s stroma, due to the rehydration process of the graft (Figure 6B). OCT showed that the epithelium and lacrimal film were intact, both the graft and cornea remained stable, the corneal edema subsided, and the transition between donor graft and recipient corneal bed could not be seen at the third month post-surgery (Figure 6C). At six months after surgery, the structure of the entire cornea remained highly homogeneous, and the graft was perfectly incorporated into the corneal tissue (Figure 6D). The thickness and collagen fiber trend of the entire cornea remained highly consistent when eyes were checked again by OCT 1 year after surgery (Figure 6E).

## 4. Discussion

In human ophthalmology, corneal opacity remains one of the most common causes of blindness [19]. Therefore, much research has been devoted to developing materials to replace the natural cornea and obtain good optical properties after surgery [20]. The development of tissue engineering has made this possible. In recent years, the engineered corneas obtained by acellular technology have been widely used in animals to evaluate this effect [16,17,21,22,23]. They have been gradually applied to various human corneal diseases, such as infectious keratitis or ulcers [18,24,25,26]. The results have shown that porcine acellular corneal tissue transplantation, with its good histocompatibility, low complications and immune rejection provided certain optical effects in the treatment of rabbit model and human corneal diseases [16,18,22,26].

In veterinary ophthalmology, FCS is a common disease in cats. In the present study, the proportion of Persian cats was overrepresented (78.69%), and almost all sequestrums were found centrally or paracentrally, which were similar findings to previous reports [8,13,15]. With the same purpose as a human corneal transplantation, after the removal of the corneal sequestrum, the ideal graft for repairing corneal defects in cats should own properties such as the maintenance of the receptor’s corneal morphology, the achievement of good transparency, the reduction of immunological rejection, and the risk of recurrence. Although various kinds of surgical methods and materials have been employed [1,8,9,10,14], these techniques often fail to achieve corneal clarity. In terms of optical effect, allogeneic or autologous corneal grafts may reduce scarring and are the best choice to repair the corneal defects of cats as a traditional treatment, but the lack of donor sources and the availability of healthy cornea that must be harvested from the same cornea limits the wide application of these materials [13,15,27]. In addition, xenogeneic amniotic membrane can also be used as one of the effective methods for the treatment of FCS, but its wide application is limited by its few sources, complex manufacturing process, and narrowed indication [12].

The BioCorneaVet^TM^ examined here is one of the ABC. The material is produced through the rapid decellularization of porcine corneal stroma using a combination of mild detergent sodium N-lauroyl glutamate (SLG) and super nuclease. Compared with traditional methods such as sodium dodecyl sulfate [28], human serum with electrophoresis [22], freeze-thaw [29], and N2 gas [30], the novel decellularization method enabled the efficient removal of xenoantigen DNA within 3 h, while retaining the ultrastructure, transparency, and mechanical properties of porcine corneas [31]. Because the sterile material is easy to store and commercially available, it can be used as a good substitute for the lamellar transplantation of feline corneal sequestrum. In the present study, BioCorneaVet^TM^ showed satisfactory corneal transparency, 100% (62/62) of eyes retained vision. The graft achieved good outcomes (0 and 1 grade) in 60/62 eyes (96.77%), even when the diameter of the corneal defect was larger than 10 mm. This may result from the good elasticity and tension of the material. Furthermore, no involvement of conjunctival tissues that would be used in CCT and conjunctival grafts surgery when we only used BioCorneaVet^TM^ in keratoplasty would not only protect the cornea and surrounding tissues from secondary damage, but also improve the transparency of the cornea post-surgery.

Complete re-epithelialization of the cornea plays an important role in the survival of a graft [32]. Such epithelialization can improve the structure of the graft, cover the wound edge to stabilize it, and provide a strong barrier function for the whole ocular surface. Our results showed that the re-epithelialization in cats was accomplished at 5–15 days (mean 7.39 days) after surgery, which was basically consistent with that in dogs [33]. However, the epithelialization could fail due to graft edema that resulted from the unmatched surface between the graft and implant bed, or from loose sutures. In that case, the graft would require resuturing. Furthermore, corneal vascularization is a non-specific reaction to inflammation and a common phenomenon of natural healing [34]. In the present study, we observed that almost all grafts had various degrees of corneal vascularization, and the dense vessels were gradually subsiding within 2–3 months after the operation, which is a similar observation to the report of Daniele et al., 2021 [33]. The role of corneal cells is crucial in the process of corneal stromal remodeling [19]. Compared with other transplantation operations (i.e., BIOSIS, conjunctival grafts, CCT, and corneal transplantation), BioCorneaVet^TM^ produced more obvious cell infiltration developed into the graft [1,2,7,13]. Their regression was observed 1–4 months after surgery as a result of the interaction between epithelial and stromal cells mediated by cytokines and receptors during corneal endogenous repair and remodeling [35,36,37,38]. Furthermore, the growth of endogenous nerves into the graft happened [39]. Although the acellular property of the graft excludes antigenic reaction in theory, immunosuppressive agents were administrated topically in order to prevent severe inflammatory reactions and obvious scarring. Further clinical research is needed to evaluate the healing effect without immunosuppressive agents.

Miscellaneous complications would determine the prognosis of the surgery. A proportional rate of implant sloughing [8], graft malacia [15] and necrosis [12], pigmentation [40], and sequestrum recurrence [2,6,11] has been reported in keratoplasty with other sources. No observations of corneal melting, pigmentation, and sequestrum recurrence were found in the present study. Focal dehiscence of wound occurred in two eyes, perhaps due to the owner’s poor collar compliance at week 2 after the operation. In addition, in this study, we found that the protrusion of five grafts was noted post-operatively, which may be related to the edema and thickness choice of the graft. Although there are multiple options in thicknesses (200, 300, 400, or 450 microns were used in the present study), that is different from those biomaterials that are superficial and need to be folded into multiple layers to match the defect [6,12,41].

Previous studies have shown that OCT could accurately evaluate corneal pathology and corneal vascularization levels, assist in keratectomy, and detect corneal morphology postoperatively [13,42,43,44,45]. In our study, OCT demonstrated that the reconstructed corneal tissue maintained high homogeneity and integrated well with the implanted cornea. Furthermore, the OCT images showed that the transitions between the graft and the recipient’s stroma were not as obvious as those of autologous keratoplasty [13], which had proved the good bio-compatibility of the graft. Meanwhile, we also had no observation of an obvious decrease in graft thickness that was regarded as a sign of collagen degradation and had been found in human use [26]. We predicted that this might be relevant with the different time schedule of OCT examination that was started from 3 months after surgery in this report. We guessed that the degraded parts would be eventually supplemented by newly formed collagen fibers, which would have no impact on the integration and light transmission of the graft.

The results have shown that the studied graft is an excellent bioengineering scaffold to promote the regenerative healing of corneal tissue and achieve the good transparency in the treatment of FCS. Some limitations in this retrospective study do exist, including but not limited to the data collection that was not completely unified and standardized, some vacancies of follow-up time, incompliance of OCT examination, and post-op photograph collection. In addition, as a retrospective study, we only discussed the effects and potential complications of ABC materials, and did not compare with other materials in the treatment of FCS. Further research should be carried out in more cases in order to compare different materials in treating FCS, evaluate the long-term results in a more accurate manner, especially the risk of sequestrum recurrence and the changes of corneal thickness by more frequent OCT examination.

## 5. Conclusions

The ABC available with different options in thickness and diameter, which is readily offered and easily preserved, is a good alternative in the treatment of FCS; the ABC not only owns a good elasticity and tension to provide a physical support, but also has less and minimal postoperative complications. All of the above features help achieve a rapid corneal healing, and satisfactory visual and cosmetic effects in FCS treatment.

## Figures and Tables

**Table 1:** Grade of transparency of the cornea.

Grade	Description	Number of Eyes
0	Ghost stromal vasculature, almost undetectable corneal opacity, and clear visualization of the posterior segment through the graft	50
1	Minimal stromal opacity and vascularization. Clear visualization of the posterior segment through the graft	10
2	Mild to moderate stromal opacity and vascularization. Visualization of the posterior segment through the graft is possible, but difficult	2
3	Marked stromal opacity and visualization allowing visualization of the anterior chamber through the graft, but not the posterior segment	0
4	Severe stromal opacity, visualization and pigmentation. Anterior chamber cannot be visualized through the graft	0

## Data Availability

The data presented in this study are available on request from the corresponding author. The data are not publicly available due to privacy.

## Supplementary Materials

The following supporting information can be downloaded at: https://www.mdpi.com/article/10.3390/ani12081016/s1, Table S1. Summary of clinical cases in the present study. RET: Re-epithelization time. MI: male intact. MC: male castrate. FI: female intact. FS: female spayed. OD: right eye. OS: left eye. C: central. P: paracentral. BSH: British shorthair. ASH: American shorthair. DSH: Domestic shorthair. Case 2 and case 11 are the same cat.
