# Supplementary material for: Lamellar Keratoplasty Using Acellular Bioengineering Cornea (BioCorneaVetTM) for the Treatment of Feline Corneal Sequestrum: A Retrospective Study of 62 Eyes (2018–2021)
Source: Animals (Basel). 2022 Apr 13;12(8):1016. doi: 10.3390/ani12081016 (PMC9026742; doi:10.3390/ani12081016)
Supplement: Supplementary file 1 [file animals-12-01016-s001.zip › Supplementary table/Supplementary table.pdf]

Supplementary data

Table S1. Summary of clinical cases in the present study. RET: Re-epithelization time. MI: male intact. MC: male castrate. FI: female intact. FS: female spayed. OD: right eye. OS: left eye. C: central. P: paracentral. BSH: British shorthair. ASH: American shorthair. DSH: Domestic shorthair. Case 2 and case 11 are the same cat.

Number of Cases	Breed	Age (months)	Gender	Affected eye	Concomitant eyelid or corneal condition	FHV-1 PCR	Sequestrum localization	Graft thickness (micron)	Graft size (mm)	Follow-up (months)	RET (days)	Grade	Post-operative complication
1	Persian	60	MI	OS	-	-	P	400	7.50	28	7	0	-
2	Persian	39	MI	OD	-	Positive	C	400	9.50	25	8	1	-
3	Persian	13	FI	OD	-	-	C	300	8.00	41	7	0	-
4	Persian	12	MI	OS	-	-	P	300	11.00	25	7	0	Partial dehiscence and resutured
5	Persian	36	FI	OD	-	Negative	C	400	10.50	24	6	0	-
6	Persian	14	FI	OD	-	-	C	400	9.50	24	7	0	Graft protrusion
7	ASH	12	FI	OS	-	-	P	300	6.00	41	6	1	-
8	Persian	24	MC	OS	-	Positive	C	400	12.00	28	9	2	-
9	Persian	8	MC	OD	-	-	P	300	6.00	41	7	0	-
10	Persian	19	MI	OS	Corneal lipid deposition	-	P	400	5.50	27	7	0	Graft protrusion
11	Persian	40	MI	OS	Corneal epithelial erosion	Positive	C	400	10.50	24	7	0	-
12	Persian	36	MI	OD	-	Positive	C	400	7.75	27	6	0	Graft protrusion
13	Persian	24	MI	OS	-	-	C	400	9.50	37	9	0	Graft protrusion
14	BSH	48	MC	OD	-	-	C	400	10.00	16	7	0	-
15	Persian	120	MC	OD	-	-	C	400	5.25	17	7	0	-
16	Persian	18	FI	OD	-	-	P	400	5.50	18	6	0	-
17	Persian	12	MI	OS	Bullous keratopathy	-	C	300	7.50	19	7	0	-
18	Persian	17	MI	OS	Corneal epithelial erosion	Positive	C	400	11.50	13	8	0	Graft protrusion
19	Persian	13	MI	OD	Corneal epithelial erosion	-	C	400	10.00	19	7	1	-
20	Persian	48	MI	OS	-	-	C	450	8.00	24	7	1	-

21	Persian	48	FS	OS	Scar after conjunctival flap	-	C	300	7.25	18	9	1	-
22	ASH	97	FS	OD	-	-	C	300	7.50	6	7	0	-
23	Persian	36	FS	OD	Eyelid entropion + Bullous keratopathy	-	C	400	10.50	14	8	0	-
24	Persian	72	MI	OS	-	-	C	400	5.00	14	7	0	-
25	Persian	24	MI	OD	Bullous keratopathy	-	C	400	5.25	12	8	0	-
26	Persian	48	MC	OD	-	-	C	400	7.75	14	7	0	-
27	Munchkin	22	MI	OS	Bullous keratopathy	-	C	300	7.25	9	7	0	-
28	BSH	101	FS	OS	-	-	C	300	6.00	14	10	0	-
29	Persian	24	MI	OD	-	-	C	400	8.00	13	7	0	Partial dehiscence and resutured
30	Persian	48	MC	OD	-	-	C	300	7.25	7	7	0	-
31	Persian	40	MC	OS	-	Positive	P	300	8.00	6	9	0	-
32	DSH	30	MC	OD	-	Positive	C	300	9.00	6	8	0	-
33	ASH	27	MC	OS	-	Negative	C	300	10.00	11	9	0	-
34	BSH	54	MC	OD	-	Negative	C	300	10.00	11	7	0	-
35	Persian	12	FS	OD	-	Negative	C	200	7.50	14	7	0	-
36	BSH	29	FS	OD	-	Negative	C	450	10.00	9	5	0	-
37	Ragdoll	6	MC	OS	-	Positive	P	400	7.50	6	7	1	-
38	Persian	74	MC	OS	-	-	C	400	10.00	8	7	0	-
39	Persian	50	MC	OS	-	-	C	300	8.00	8	7	0	-
40	Persian	72	MC	OS	-	-	C	400	9.00	10	7	0	-
41	Persian	53	MC	OD	-	-	C	400	10.00	13	6	0	-
42	Persian	14	MC	OS	-	-	C	300	12.00	4	7	0	-
43	Persian	31	MC	OS	Bullous keratopathy	Negative	C	300	7.50	13	7	0	-
44	Persian	52	MC	OD	-	-	C	300	7.00	13	10	0	-
45	ASH	27	MC	OS	Eyelid entropion	-	P	400	6.50	11	15	0	-
46	Persian	36	MC	OS	-	Positive	C	450	10.00	12	6	0	-
47	Persian	54	MC	OS	-	-	P	300	8.00	11	8	0	-
48	Persian	24	FS	OS	-	Positive	C	300	6.50	12	10	0	-
49	Persian	25	MC	OS	-	-	C	300	7.00	11	7	0	-
50	Persian	39	FS	OD	-	-	P	300	8.50	10	6	0	-
51	Persian	24	MC	OD	Bullous keratopathy	Negative	C	400	7.00	6	6	0	-
52	Persian	24	FS	OD	-	-	C	400	7.00	6	6	0	-

53	Persian	24	FS	OD	Bullous keratopathy	Positive	C	300	9.00	12	7	1	-
54	Persian	60	MI	OD	Corneal epithelial erosion	Negative	C	400	7.75	5	7	0	-
55	Persian	34	MI	OD	Corneal epithelial erosion + Bullous keratopathy	Positive	P	300	6.00	5	5	0	-
56	Persian	66	FS	OD	Corneal melting	Negative	C	400	10.25	4	7	1	-
57	Persian	14	FI	OD	-	-	P	300	7.50	4	7	1	-
58	Persian	53	MC	OD	-	-	P	300	9.00	3	7	2	-
59	BSH	18	FI	OD	Bullous keratopathy	Negative	C	300	9.75	6	8	0	-
60	Persian	14	MI	OS	-	-	C	400	5.50	6	8	0	-
61	BSH	27	MI	OS	Eyelid entropion	-	P	450	8.50	7	9	1	-
62	Persian	21	MI	OS	Eyelid entropion	-	C	300	8.00	6	7	0	-
